# A comprehensive update of extracutaneous involvement of mycosis fungoides: A narrative review of literature

**DOI:** 10.1097/MD.0000000000042279

**Published:** 2025-05-09

**Authors:** Lingxi Liu, Xiaoyan Zhang

**Affiliations:** a Department of Dermatology, West China Second University Hospital, Sichuan University; Key Laboratory of Birth Defects and Related Diseases of Women and Children (Sichuan University), Ministry of Education, Chengdu, Sichuan, China.

**Keywords:** clinical manifestations, extracutaneous involvement, mycosis fungoides, review, update

## Abstract

Mycosis fungoides, the most prevalent subtype of cutaneous T-cell lymphoma, primarily targets areas of the skin that of not exposed to sunlight. Nevertheless, it can involve extracutaneous tissues at any disease stage, especially during its advanced phases. Research suggests that all extracutaneous organs might be susceptible, though such cases are more commonly reported in autopsy studies and less so in clinical setting. The most recent comprehensive review of such extracutaneous manifestations in mycosis fungoides occurred almost a decade ago. With the aim of enhancing clinicians’ understanding of these extracutaneous manifestations to improve diagnostic accuracy and patient outcomes, this updated review provides a current synthesis of the rare instances of extracutaneous involvement in mycosis fungoides.

**Key Summary Points**

Mycosis fungoides primarily affects the skin in non-sun-exposed areas; however, it can also spread to extracutaneous organs, especially in the advanced stages of the disease.

The involvement of internal organs, as estimated from clinical progression, appears to be lower than what autopsy studies suggest.

Patients with extracutaneous mycosis fungoides generally face a poor prognosis, although early detection and intervention can alleviate clinical symptoms and reduce tumor burden.

In patients with a history of mycosis fungoides, the presence of extracutaneous manifestations should prompt consideration of potential involvement of other organs.

Advanced cutaneous manifestations, extensive lymph node involvement, large cell transformation, or a shift in immunophenotype from CD4+ to CD8+ are associated with an increased risk of progression to extracutaneous involvement.

## 1. Introduction

Cutaneous T-cell lymphomas (CTCL) are the most common primary cutaneous lymphomas, with mycosis fungoides (MF) being the dominant subtype. MF accounts for approximately half of all lymphomas that originate in the skin.^[1]^ Despite its commonality within CTCL, MF is considered a rare disease due to the low overall incidence of CTCL.^[2]^ There are several variants of MF, including folliculotropic MF, pagetoid reticulosis, and granulomatous slack skin.^[2]^ These variants exhibit unique clinical, histological, and hematological characteristics, along with different prognoses. Consequently, they were excluded from the analyses conducted in this review.

MF is characterized by clonal T-helper cells and exhibits a low degree of malignancy, typically presenting as a chronic skin disorder. The disease progression typically follows a predictable pattern, which includes 3 phases: an erythematous or eczematous rash, infiltrated plaques, and cutaneous tumors.^[3,4]^ The majority of patients diagnosed with MF primarily exhibit early-stage manifestations, characterized by persistent, erythematous, scaly patches or plaques, predominantly affecting the limb girdle areas that receive less sun exposure. As the disease progresses, a subset of patients may develop the late-stage symptoms, marked by the emergence of skin tumors, erythroderma, and potential infiltration of lymph nodes and internal organs.^[1,5]^ The rate of disease progression varies significantly, allowing for the simultaneous presence of patches, plaques, and tumors in different skin areas, as well as the possibility of presenting with extracutaneous involvement initially.^[6]^

The staging systems for CTCL are based on the tumor, lymph node, and metastasis (TNM) classification originally proposed by Bunn and Lamberg in 1979.^[7]^ This system was later revised in 2007 by the International Society for Cutaneous Lymphomas (ISCL) and the European Organization for Research and Treatment of Cancer (EORTC) to incorporate a blood stage, leading to the development of the TNMB classification.^[8]^ This classification incorporates multiple factors including the extent of body surface involvement (T1 or T2), the nature of skin manifestations (patches/plaques or tumors designated as T3, or erythroderma as T4), the presence or absence of lymph node (N0-N3) or visceral organ involvement (M0-M1), and the extent of blood tumor burden (B0-B2).^[8]^ Therefore, the original 10 TNM categories have expanded to 20 TNMB categories, covering 6 skin stages, 7 nodal stages, 2 metastatic stages, and 5 blood stages.^[8–10]^ These categories are employed to define 9 stages, ranging from IA to IVB.^[5,9]^ Stages IVA and IVB reflect extracutaneous lymph node or visceral involvement.^[11]^

The vast majority of current research has focused on the early stages of diseases that solely involve the skin (stages I to III), with limited attention given to extracutaneous disease (stage IV). MF has been shown to affect lymph node and all internal organs, although evidence primarily comes from autopsy series, with few clinical studies and case reports available. In 1914, Paltauf et al^[12]^ documented instances of MF affecting nerves and internal organs. Liechti^[13]^ provided a brief overview of case studies involving MF with visceral manifestations in 1927. Subsequently, a comprehensive analysis conducted in 1972 on 144 patients with MF focused on survival rates, prognostic indicators, treatment responses, and postmortem findings.^[14]^ In 1974, 2 research groups published findings from autopsy series of patients who had succumbed to MF, detailing the extracutaneous manifestations of the disease.^[15,16]^ Later research using advanced methodologies demonstrated the presence of extracutaneous MF in a significant number of patients, even in the early stages of the illness.^[17]^ Among the extracutaneous organs affected, lymph nodes were found to be the most commonly involved, with varying degrees of visceral participation observed in different studies. The most frequently affected visceral sites include the lymph nodes, liver, spleen, lungs, oral cavity or pharynx, gastrointestinal tract, and central nervous system.^[10,11,18,19]^

The extracutaneous involvement in patients with MF represents a distinct clinicopathological phase in the disease’s natural progression, rather than a transformation of MF into a different histological subtype of lymphoma.^[15]^ The likelihood of progression to extracutaneous involvement is higher in patients with more advanced skin manifestations of the disease. As the T-classification level increases, so does the risk of progression to extracutaneous disease.^[6]^ The presence of extracutaneous involvement is recognized as a significant independent prognostic factor, alongside age, gender, T-cell classification, and response to initial treatment.^[1,10]^ A correlation has been observed between the severity of clinical symptoms and the extent of visceral involvement.^[14]^ Once extracutaneous dissemination occurs, the progression to mortality is rapid. The 5-year overall survival rates vary widely, from nearly 100% for stage IA to less than 10% for stage IVB.^[10,19]^

Various treatment options, such as single or combination chemotherapy and molecular-targeted therapy, are used for patients with MF who present with extracutaneous involvement. However, only a limited number of patients achieve complete remission (CR) with the initial treatment regimen following their diagnosis of extracutaneous disease.^[14,20,21]^

Despite the generally poor prognosis for patients with extracutaneous involvement, early detection and prompt treatment can significantly improve both prognosis and clinical symptoms. Currently, the likelihood of extracutaneous involvement is significantly underestimated, often leading to frequent misdiagnoses or missed diagnoses. This issue stems from a lack of sufficient knowledge among clinicians about extracutaneous involvement. Notably, the most recent comprehensive review on this topic is nearly a decade old. Consequently, a literature search was conducted to provide an updated and thorough review on extracutaneous involvement of MF. This review is based on previously conducted studies and does not contain any new studies with human participants or animals performed by any of the authors.

## 2. Extracutaneous involvement of MF

### 2.1. Lymph node

Patients with MF may exhibit regional lymphadenopathy, which is typically reactive in the early stages, with potential lymph node involvement as the disease progresses. Lymph node involvement, characterized by the partial or complete effacement of lymph node architecture by malignant cells, is often the earliest and primary extracutaneous manifestation in patients with MF.^[22,23]^ This presentation exhibits morphologic variability among cases.^[24]^ Peripheral lymph node involvement is more prevalent than central lymph node involvement, with inguinal/femoral lymph node involvement being 2 to 3 times more common than axillary node involvement.^[17]^ Lymph node involvement is a significant factor in determining therapeutic strategies, patient response, and overall survival rates. Patients with enlarged lymph nodes have a poorer prognosis compared to those without lymphadenopathy, even after adjusting for the presence of skin involvement.^[24,25]^ Patients diagnosed with early-stage disease typically exhibit a favorable prognosis, with survival rates ranging from 10 to 35 years. In contrast, those presenting with nodal involvement have a markedly reduced survival rate, averaging only 13 months.^[9]^ Furthermore, patients with definitive histopathological confirmation of lymph node involvement exhibit a worse prognosis than those without obliterated lymph nodes, such as patients with dermatopathic lymphadenopathy or other reactive alterations.^[24,26]^ Patients presenting with significant lymphadenopathy are more likely to exhibit visceral involvement. Patients with lymph node-only metastasis have better survival outcomes compared to those with metastasis to other sites.^[23]^

Current staging and diagnostic guidelines do not require biopsy of clinically normal lymph nodes. However, enlarged lymph nodes that display hardness, irregular morphology, clustering, or restricted mobility should be further examined.^[27]^ In cases where multiple abnormal lymph nodes are identified, priority for biopsy should be given to cervical, axillary, and inguinal lymph nodes, with cervical nodes being more suggestive of lymphomatous involvement.^[8,24,27]^ Furthermore, the biopsy should target either the largest lymph node draining the affected dermatological region or the node with the highest standardized uptake value as determined by FDG-PET imaging.^[5,28,29]^

Accurate assessment of lymph node involvement in patients with MF especially in the initial phases, remains challenging. T-cell clonality analysis is a crucial tool for differentiating between benign dermatopathic lymphadenitis and early LN involvement.^[22]^ The consistent identification of identical dominant T-cell clones in both skin and lymph node biopsies via Genescan analysis across various cases suggests that these dominant T-cell clones in lymph nodes stem from cutaneous tumor cells. This observation indicates disease progression rather than a physiological recirculation of T-cells.^[22]^ Patients without clonal T-cell involvement in lymph nodes tend to have a significantly longer survival period than those with clonal lymph node involvement.^[22]^ Furthermore, diagnostic imaging techniques such as ultrasound, computed tomography (CT), positron emission tomography (PET), and magnetic resonance imaging (MRI) can support in the diagnostic process.

### 2.2. Nervous system

The infiltration of nerves by neurotropic neoplastic cells in cases of either unidentified or known hematologic malignancy, is clinically referred to as neurolymphomatosis (NL).^[30]^ NL is predominantly associated with non-Hodgkin lymphoma (NHL), accounting for 90% of cases, and acute leukemia, which makes up the remaining 10%.^[30]^ In patients newly diagnosed with MF, the highest risk of developing neurological involvement occurs within the initial years following diagnosis and correlates with the early stages of the disease.^[31]^

#### 2.2.1. Central nervous system

The presence of CNS involvement in patients with MF has been sporadically documented, with reported frequencies ranging from 1.3% to 15%, often detected postmortem.^[31]^ This involvement has been observed in autopsy series as intracerebral tumor masses, leptomeningeal infiltration, or cerebral hemorrhages.^[32]^ Among these, meningeal infiltration was identified as the most prevalent pathological feature.^[33]^ When metastasis to the CNS occurs, more than half of the affected patients also present with involvement of lymph nodes and/or other visceral systems, including the pancreas, liver, spleen, nasopharynx or oropharynx, kidney, and eye.^[34]^ CNS invasion can also occur independently of lymphadenopathy and may even represent the sole manifestation of metastatic disease.

Central neurologic involvement in MF typically emerges during the advanced stages of the disease, characterized by symptoms such as fluctuating higher cognitive functions and rapid onset of cranial nerve dysfunction.^[31]^ The clinical manifestations are highly variable. Gait instability and weakness are identified as the most prevalent CNS symptoms, followed by confusion, amnesia, and slowed cognitive processing.^[34]^ Other reported symptoms include lethargy, unsteady gait, speech difficulties, paresthesia, coma, and flaccid paralysis.^[20,35,36]^ According to a literature review, ocular/orbital disease is highly concordant with CNS involvement in lymphoma and other tumors.^[37,38]^ Ocular manifestations, including diplopia, blurred vision, blindness, nystagmus, hemianopia or quadrantanopia, and papillary edema, have been reported to be relatively common among patients with CNS involvement in MF.^[34]^

The diagnosis of CNS involvement is established through clinical presentation, cerebrospinal fluid (CSF) analysis, biopsy, or autopsy. Imaging studies can reveal abnormal findings in nearly all patients. MRI detected CNS abnormalities in 91% of cases, whereas CT scans identified CNS lesions in 74% of cases.^[34]^ Examination of the CSF can reveal an increase in Sézary cells or lymphocytes, elevated protein and glucose levels, and increased opening pressure.^[39,40]^ Only a small proportion of patients exhibited no significant abnormalities in their CSF. Preston et al^[36]^ have proposed that imaging alone may be adequate for diagnosing certain patients with CNS involvement.

The prognosis for patients with CNS involvement is generally poor, displaying a high mortality rate. In a review series of MF patients with CNS involvement, the observed survival rate stood at 12%, with a median time to death of 9 weeks following the diagnosis of CNS involvement.^[34]^ Immunophenotype switching from CD4 + to CD8 + may serve as an early indicator of aggressive disease, thereby necessitating thorough systemic and neurological evaluations for the early detection of MF dissemination and the prompt initiation of treatment.^[41]^ Large cell transformation (LCT) is associated with an increased risk for CNS metastasis,^[36,39]^ and a more recent studies indicated that 24% of cases with LCT occurred prior to CNS involvement in CTCL.^[34]^ Stein et al^[31]^ developed a risk calculator to assess the probability of CNS involvement, identifying high-risk patients as those with 2 or more of the following factors: T stage ≥ 3, N3, M1, or B1. Given the significant morbidity and mortality associated with CNS involvement, clinicians should remain vigilant for this potential manifestation of metastatic disease, notwithstanding its rarity.^[36]^

Although the prognosis is generally poor, certain patients have demonstrated clinical benefit from CNS-directed therapies, including radiation, surgery, and high-dose methotrexate (MTX).^[36,42]^ Notably, these treatments can also provide symptomatic palliation, which is crucial for maintaining patient quality of life, even in the absence of a survival benefit.^[36]^ The efficacy of low-dose prophylactic cranial radiation remains uncertain, yet urgent palliative treatment is necessary for cases of overt neurologic involvement.^[31]^ Although studies have demonstrated a poorer prognosis in untreated patients compared to those who receive treatment, patients who achieve remission generally require less medication than those who do not survive.^[34]^ Consequently, management strategies should be tailored according to the disease burden, clinical presentation, and functional status of each patient. Where feasible, single-agent chemotherapy and radiation should be employed to mitigate the risks associated with overaggressive multi-agent treatments, which have not been shown to enhance survival outcomes.^[34]^

#### 2.2.2. Peripheral nervous system

Peripheral nervous system (PNS) involvement in MF is exceedingly rare and can occur through direct compression or infiltration,^[33,43,44]^ non-infiltrative mechanisms,^[45]^ or transformation into large T-cell lymphoma.^[44]^ Direct nerve infiltration is characterized by endoneurial fibrosis, reduced myelinated fibers, and acute axonal degeneration.^[33]^ Rappaport and Thomas identified that atypical lymphocytic infiltrates in peripheral nerves tend to exhibit a predilection for perineural sheaths, with subsequent invasion of the epineurium.^[16]^ Such infiltrations have been associated with severe pain and motor paralysis. A case study reported a rare autoimmune, chronic, mixed axonal-demyelinating, sensorimotor polyradicular neuropathy in MF, suggesting that peripheral neuropathy can occur without direct tumor invasion of nerves.^[45]^

The onset of neurological complications in patients with MF appears to be closely associated with the stage of skin disease, such as the presence of tumor lesions and erythroderma.^[46]^ Nonetheless, PNS involvement can also serve as the sole indicator of extracutaneous involvement.^[47,48]^ The clinical manifestations of PNS involvement exhibit considerable variability, primarily contingent on the extent of nerve involvement.^[47]^ In the initial stages, symptoms are frequently subtle and may go unrecognized by the patient. However, as the disease advances, patients may experience a spectrum of neurological deficits, including pain, plexopathy, foot drop, mononeuritis multiplex, cranial nerve palsies, and radiculopathy, depending on the specific nerves affected.^[45,47,48]^ Although the progression of the disease is variable, it is generally indolent and confined to a single nerve.^[47]^ Additionally, 26% of patients with PNS involvement also exhibit CNS involvement.

Imaging studies, such as MRI and PET-CT, are highly valuable in clinical practice. MRI can detect abnormalities in nearly 80% of patients; however, its findings lack specificity. When combined with MRI results, PET-CT can help identify the most suitable site for a biopsy. In cases where imaging and CSF results are inconclusive, a nerve biopsy may be considered if the potential benefits outweigh the risks.^[30]^

Currently, there is no established protocol for treating PNS involvement. The reported therapeutic strategies include systemic chemotherapy, systemic or intrathecal chemotherapy, radiation therapy, and brain irradiation, either as monotherapies or in combination.^[33,44,45]^ Although clinical improvement has been observed in some treated individuals, the overall prognosis remains unfavorable.

### 2.3. Gastrointestinal tract

MF can occasionally affect every segment of the gastrointestinal (GI) tract, from the oral cavity to the rectum, as well as the pancreas and biliary tract. When the disease spreads from the skin to the digestive tract, it primarily affects the stomach, small bowel, and pancreas.^[49]^

#### 2.3.1. Oral cavity

Oral MF is a rare condition, with fewer than 100 reported cases to date. A study conducted by Sirois et al documented an incidence of oral lesions below 1% among 824 patients diagnosed with MF over a 25-year span.^[50]^ Although there have been rare instances where oral lesions precede cutaneous lesions, the majority of patients have a pre-existing MF diagnosis. Only 4 cases have been thoroughly documented wherein oral lesions were the initial presentation of MF.^[51–53]^ A review of existing literature indicates a higher prevalence of oral MF in males.^[53,54]^ Oral MF has impacted a wide range of intraoral sites, with lesions identified at multiple locations in approximately 50% of the cases.^[53]^ The most common sites of oral MF involvement are the tongue, palate, and gingiva.^[53–55]^ Oral MF can exhibit a wide range of clinical presentations, primarily characterized by erythematous and/or ulcerated plaques, tumors, and nodules.^[51,54]^ Additionally, it may present as leukoplakia or pseudomembranous changes.^[53,54]^ Other clinical manifestations include asymptomatic soft swellings, indurated plaques, papules, and erosions.^[56]^ Lesions on the tongue may also result in the loss of lingual papillae.^[51]^ The symptoms associated with oral MF are often nonspecific, with dysphagia and pain being the most frequently reported.^[54]^

Early detection of oral involvement is crucial as it may enhance of treatment efficacy. Managing oral lesions presents a greater challenge compared to managing cutaneous lesions.

Comparative analyses between cutaneous and oral MF typically reveal similar histological characteristics. The predominant immunophenotype of oral MF is characterized by helper T-cells (CD4+, CD8-), with a secondary prevalence of a dual-positive phenotype (CD4 + CD8+).^[54]^ A cytotoxic T-cell phenotype (CD8+, CD4-) is rarely observed. To date, only 7 cases of oral MF with documented LCT has been reported.^[3,54,57–59]^

The observation that patients with MF exhibiting oropharyngeal metastasis also had a higher incidence of concurrent metastasis to other organs is noteworthy, suggesting a potential correlation between oropharyngeal involvement and a more aggressive disease phenotype.^[23]^ Mortality was observed approximately one year following the initial occurrence of the oral lesions.^[54]^ This survival rate is inferior to that observed in patients with erythroderma, lymphadenopathy, or tumoral lesions, and is comparable to those with visceral involvement.^[15,60]^ Consequently, oral involvement in MF has been regarded as an indicator of poor prognosis and advanced disease. Fortunately, with advancements in current treatment methods, both the survival rate and lifespan of patients with oral MF have seen significant improvements. Bassuner et al^[57]^ reported a complete response lasting 7 years in a patient with oral MF and LCT, who was treated with local electron beam therapy and maintenance therapy with oral Bexarotene.

#### 2.3.2. Esophagus

Involvement of the esophagus in MF is exceedingly rare, with the majority of documented cases being identified postmortem.^[61]^ The incidence of esophageal involvement detected during autopsy is notably low. Rappaport and Thomas analyzed 45 autopsied cases of MF and observed esophageal involvement in only 4 instances.^[16]^ Similarly, Epstein et al^[14]^ reported esophageal involvement in only 7 out of 86 postmortem cases. Consequently, esophageal involvement is generally regarded as an incidental finding during autopsy. Notably, in 1990, Kim et al^[62]^ reported the first case of esophageal MF confirmed by biopsy prior to death. To date, only a few cases have been documented with comprehensive clinical and pathological information in the English literature.

Nearly all patients exhibited cutaneous manifestations of MF before the development of esophageal lesions. Esophageal MF typically presents as an ulcerative tumor.^[61,63]^ The associated symptoms include dysphagia, sore throat, odynophagia, hoarseness, and reflux,^[61,62]^ which are frequently attributed to local compression by the tumor and mucosal damage. In certain instances, esophageal involvement occurred concurrently with involvement of the oropharynx, pharynx, and larynx, suggesting a proximal-to-distal progression of the disease.^[63]^ Kim et al^[62]^ documented a case of a patient presenting with conscious dysphagia, where endoscopic examination revealed no esophageal abnormalities. However, esophageal MF was confirmed through a biopsy. The authors postulated that the symptoms could be attributed to the involvement of sensory nerve fibers within the esophagus.

#### 2.3.3. Gastrointestinal tract

In most cases, GI lymphomas are classified as extranodal NHLs, predominantly characterized by proliferating B cells.^[64,65]^ Only 1.5% to 4% of gastrointestinal NHLs are identified as the peripheral T-cell lymphoma histological subtype. GI tract involvement has been documented in 9-40% of autopsied cases. MF gastrointestinal involvement can manifest either as a primary condition or as a secondary consequence of dissemination from cutaneous disease. Primary gastrointestinal forms of MF are exceedingly rare.^[66]^

Sporadic studies have documented MF involvement in the stomach. Scali et al^[67]^ reported on a 65-year-old female patient who presented with gastrointestinal symptoms, including gastric pain, a decade after being diagnosed with dermatological manifestations of MF. Gastroenteroscopic examination revealed an ulcerative nodule in the gastric mucosa, and subsequent gastric biopsy results indicated T-cell lymphoma. To our knowledge, this is the only documented case of biopsy-proven MF with gastric metastasis. Another case involved a patient with advanced MF who experienced nocturnal epigastric pain; a postmortem examination revealed an 8 cm ulcer in the gastric cardia, which histological analysis confirmed as MF.^[68]^

There have been several isolated case reports documenting intestinal involvement in MF to date, the cases indicate that the small intestine is the most frequently affected gastrointestinal site in MF.^[69]^ Small intestinal involvement predominantly appears in the advanced stages of the disease; however, instances of early progression have also been noted. One case was involved a patient with stage ⅠA MF who initially responded well to treatment; however, the disease rapidly progressed, leading to small intestine involvement and subsequent intestinal obstruction within 3 months.^[69]^ The prognosis for patients with small intestine involvement is generally poor, with a majority succumbing within 6 months of diagnosis.^[3]^ Additionally, a rare instance of MF involving the duodenal papilla has been reported by Gomez Venegas and coworker.^[49]^ Two instances of rectal MF have been documented, one verified through biopsy and the other identified postmortem.^[68,70]^

Typically, gastrointestinal tract involvement progresses insidiously, manifesting with nonspecific symptoms such as nausea, vomiting, abdominal pain, diarrhea, and constipation.^[3,67,71]^ The primary complications include hemorrhage, obstruction, perforation, malabsorption, and weight loss, which may become life-threatening in severe instances.^[66,68,69]^ Gastrointestinal perforation is a well-documented complication of lymphoma involvement in the GI tract.^[3,72]^ The most common site of perforation is the small intestine.^[72]^ Perforation typically manifests as an early indicator of gastrointestinal involvement rather than as a consequence of antineoplastic therapy. Additionally, treatment may lead to the formation of perforations, generally emerging within a few weeks of commencement. Notably, the risk of perforation originating from the tumor itself may be higher.^[3]^ It is imperative for clinicians to exercise vigilance in distinguishing these conditions.

#### 2.3.4. Pancreas

Among the visceral involvement of MF, pancreatic infiltration is observed in 0-40% of autopsied cases.^[14–16,73]^ However, clinically significant involvement of the pancreas by MF appears to be exceedingly rare. To date, only 3 patients with MF have been reported with clinically detectable pancreatic metastases.^[74–76]^ Ceriolo et al^[74]^ hypothesized that a potential mechanism for pancreatic metastasis in MF involves the expression of the CCL27 chemokine by glucagon-secreting cells within the pancreatic islets. This chemokine may attract metastatic MF cells that express the corresponding receptor, CCR10.

The primary manifestations include abdominal pain and jaundice, although fatigue and weight loss may also be present. In one case, a patient developed a palpable abdominal mass. Imaging studies conducted on all 3 patients identified a mass situated at the head of the pancreas. According to the autopsy findings reported by Rappaport et al,^[16]^ 12 out of 29 examined pancreases (41%) exhibited microscopic involvement without the presence of gross tumors, indicative of a more infiltrative pattern. The prognoses of the 3 patients varied: in one case, biliary obstruction was resolved, but skin lesions deteriorated due to progressive lymphoma; another patient succumbed to septic complications; and the third patient remained alive after a 3-year follow-up period.

It is crucial to distinguish pancreatic involvement in MF from other pancreatic condition such as primary pancreatic tumors, autoimmune pancreatitis, and neuroendocrine tumors.^[74,77]^ Endoscopic ultrasound aids in differentiating between primary and secondary pancreatic tumors, with pancreatic metastases typically displaying well-defined margins.^[78]^ The main diagnostic indicators include the patient’s history of MF, along with histomorphological analysis, immunohistochemical profiling, and T-cell receptor gene rearrangement studies from both pancreatic and skin biopsy specimens.

#### 2.3.5. Liver and gallbladder

Hepatic involvement has predominantly been documented in autopsy studies, illustrating its occurrence as part of systemic multiorgan dissemination. The reported rates of liver involvement demonstrate significant variability across studies. For instance, Tang et al^[23]^ documented 35 cases of CTCL with extracutaneous metastases, of which 12 were MF cases, and only one MF patient developed liver metastases. Cyr et al^[73]^ found liver involvement in 5 out of 42 autopsies (12%). Rappaport et al^[16]^ reported 42 cases undergoing autopsy, liver involvement was evident in 17 cases (41%), with 11 (65%) showing gross tumors. Additionally, a large study involving 120 patients, with 86 undergoing autopsies, reported liver involvement in 35 cases (41%).^[14]^ In another study, hepatic involvement was observed in 13 out of 15 autopsies, corresponding to an incidence rate of up to 87%.^[15]^

Earlier autopsy studies have documented gallbladder involvement in case of MF, although with a low incidence.^[14,15]^ There have been only a limited number of reported cases where the biliary tract serves as the primary site of involvement. Albukerk et al^[79]^ describe a patient with MF who developed jaundice and pruritus due to portal tract infiltrates of MF, which led to the destruction of small interlobular bile ducts. Notably, the large intrahepatic and extrahepatic bile ducts were not affected in this patient. Madsen et al^[80]^ documented a case involving a patient with a prior diagnosis of cutaneous MF who presented with jaundice, diarrhea, and weight loss. Despite undergoing endoscopic examinations and biopsies of the biliary tree and liver, the etiology of the obstructive symptoms remained undetermined. Ultimately, surgical resection of the gallbladder and extrahepatic ducts was performed. Histopathological examination and T-cell receptor (TCR) gene rearrangements revealed findings consistent with those previously observed in the skin. Additionally, there was a report of isolated pancreatic involvement by MF which also caused biliary obstruction due to external compression of the intrapancreatic segment of the common bile duct.^[76]^ These reports underscore the importance for clinicians to remain vigilant regarding the potential involvement of the bile duct in patients with MF who present with jaundice.

#### 2.3.6. Spleen

The spleen is recognized as one of the most commonly affected extracutaneous organs in MF. Spleen involvement was observed in more than half of the patients according to a study conducted by Rappaport et al.^[16]^ Epstein et al^[14]^ reported that the incidence of spleen involvement was second only to lymph node involvement, reaching up to 50%. Another study indicated that spleen involvement could be as high as 80%.^[15]^ Splenomegaly identified through physical examination or imaging studies is classified as a visceral disease, irrespective of histological confirmation.^[27]^ Most cases of splenic involvement occur in an interstitial and diffuse manner, with no circumscribed visible tumor masses.^[16,81]^ An enlarged spleen occasionally displayed multiple small or large nodules, resembling other malignant lymphomas in gross appearance. It is crucial to acknowledge that splenic involvement may manifest in the early stages of the disease.^[81]^

### 2.4. Respiratory system

#### 2.4.1. Larynx

Laryngeal involvement in MF is exceedingly rare. Among 32 postmortem cases, laryngeal involvement was confirmed in only 2 instances, both of which exhibited gross tumors.^[16]^ In a separate autopsy study, laryngeal involvement was observed in merely 3% (3 out of 86) of patients.^[14]^ Prior to death, only 10 cases of laryngeal involvement have been documented in the English literature.^[82–86]^ Although laryngeal involvement is primarily regarded as a visceral dissemination of MF, there have also been reports of primary extracutaneous lesions of the larynx.^[87,88]^

Laryngeal MF exhibits a predilection for the arytenoids, aryepiglottic folds, and the laryngeal surface of the epiglottis.^[89]^ It can also involve the pyriform sinus, as well as the false and true vocal cords.^[89,90]^ Patients with laryngeal MF usually experience nonspecific symptoms such as dysphagia, hoarseness, dysphonia, a globus sensation, cough, throat tightness, and airway obstruction, with dysphagia and hoarseness being the most common. Therefore, when assessing patients with MF who present with hoarseness or dysphagia, it is crucial to consider the possibility of laryngeal involvement in the differential diagnosis. Other conditions to rule out include fungal infections, tuberculosis, vocal cord nodules or polyps, squamous cell carcinoma, and multiple myeloma.^[82]^

Laryngeal involvement, similar to other forms of visceral dissemination, typically manifests in the terminal stages of the disease, resulting in a poor prognosis. Most reported cases have lead to mortality within a short duration.^[82,86,89]^ In a particular case, a patient with laryngeal involvement after liver transplantation found relief from both cutaneous and laryngeal symptoms by reducing the dosage of immunosuppressant and administering oral corticosteroids.^[83]^ Another patient experienced significant improvements in symptoms and quality of life following surgical laser therapy; however, the symptoms recurred 5 months after the surgery. Notably, the laryngeal rash completely resolved after the administration of palliative chemotherapy.^[85]^

#### 2.4.2. Lung

The lungs are frequently affected sites in cases of visceral involvement, although pulmonary involvement is often identified postmortem.^[16,91,92]^ Disseminated pulmonary MF has been documented in only a limited number of case reports. Typically, these cases exhibit a rapid and fatal progression of the disease. Baser et al^[93]^ conducted a retrospective analysis of 710 patients with MF, identifying lung involvement in 7 patients (less than 1%). Their findings also indicated that patients with lung involvement exhibited shorter overall survival and reduced survival duration from the onset of lung manifestations.^[93]^ Clinical manifestations include cough, fever, dyspnea, hemoptysis, and expectoration.

Radiographic manifestations of pulmonary involvement in MF are not specific and have been documented in a few case reports. These manifestations include peribronchovascular nodules, ground-glass opacities, bilateral reticulonodular infiltrates, bilateral and diffuse interstitial opacities, patchy areas of consolidation, isolated pleural effusion, and hilar and mediastinal lymphadenopathy.^[92–97]^ Additionally, the presence of multiple progressing nodules or a solitary nodule has also been observed.^[93]^ High-resolution CT is useful for diagnosing pulmonary involvement in MF.^[94,95]^

Patients with advanced MF often display compromised immune function, particularly in T-cell-mediated immunity. This immunosuppression can be worsened by oncological treatments, thereby increasing the susceptibility of MF patients to pneumonia. From clinical and radiographical perspective, pulmonary involvement by MF may resemble pneumonia, making it crucial to differentiate between the 2 conditions since their therapeutic approaches significantly diverge.^[93,98]^ Typically, patients diagnosed with pneumonia present with pronounced respiratory symptoms and radiologic evidence of opacities. In contrast, those with pulmonary involvement by MF usually show minimal respiratory symptoms and exhibit progressive nodular disease on chest radiographs. Moreover, if clinical symptoms worsen despite adequate antibiotic therapy, it might be necessary to consider CT-guided needle biopsy, bronchoscopy, or even a wedge biopsy to ascertain the diagnosis.^[99]^

The definitive diagnosis of lung involvement requires histopathology. Tumor cells exhibit highly indented or cerebriform nuclei surrounded by a thin rim of clear cytoplasm. As the disease progresses, there is an increase in the number of large tumor cells, characterized by large cerebriform pleomorphic or blastoid nuclei. These may be mistaken for peripheral T-cell lymphoma, not-otherwise-specified (PTCL-NOS).^[99]^ Although T-cell receptor gene rearrangement studies might appear useful for elucidating the relationship between pulmonary and skin lesions, the clonal heterogeneity of MF has been well-documented, complicating such analyses.^[100]^ Next-generation sequencing technologies could potentially offer greater insight into these clonal relationships.^[101]^

### 2.5. Heart

Cardiac tumors constitute a heterogeneous group, categorized into primary and secondary tumors. Secondary cardiac tumors are more prevalent than primary cardiac tumors, comprising the majority of cardiac malignancies.^[102,103]^ Approximately 14% of secondary cardiac tumors are lymphoma deposits, predominantly NHL.^[104]^ Cardiac metastases may also be present in patients with MF, typically identified incidentally during autopsy.^[105]^ Various studies have documented differing proportions of cardiac involvement in patients with MF. Epstein et al^[14]^ reported that, among 86 patients who underwent autopsy, cardiac involvement was observed in 17% (15/86) of cases. Specifically, 5 cases involved the pericardium, 6 involved the myocardium, and the remaining cases did not specify the location of involvement. Another investigation identified cardiac involvement in 38% (12/32) of the patients studied.^[16]^ In contrast, Cyr et al^[73]^ found that only 2 out of 42 postmortem patients (5%) exhibited cardiac involvement.

To date, there have been no reported cases of where cardiac involvement is the primary manifestation of MF; all cases have involved patients with a prior history of cutaneous MF. The majority of these patients present with no discernible clinical signs or symptoms, making antemortem diagnosis challenging. Symptomatic myocardial involvement remains rare. Various factors, including tumor location, size, invasion, growth rate, and friability, influence the clinical presentation of cardiac metastases.^[104]^ Several case reports have documented the clinical manifestations of cardiac involvement, such as dyspnea, dizziness, lower limb edema, radiating left chest pain, arrhythmia, fluctuation in blood pressure, and cardiac arrest.^[105–108]^ MF may infiltrate the myocardium and disrupt the impulse conduction system, potentially resulting in arrhythmia and cardiac arrest. Additionally, MF can induce cardiac tamponade through epicardial infiltration, leading to cardiac arrest. There has been a reported case of MF presenting as a cardiac mass in the right atrium, which result in symptoms of congestive heart failure.^[105]^ Cardiac involvement in MF typically manifests as myocardial infiltration, and the presentation of a mass is relatively uncommon.^[105]^ Research suggests that MF uniquely induces rhythm disturbances, contrasting with other lymphomas such as Hodgkin lymphoma, which more commonly exhibit symptoms related to pericardial masses.^[109,110]^

While numerous cases of MF cardiac metastasis may have been clinically unrecognized in the past, advances in imaging techniques, such as transesophageal echocardiography (TEE), echocardiography, CT, and MRI, have significantly improved the detection rate.^[104]^ Furthermore, hematological abnormalities, including elevated levels of lactate dehydrogenase, B-type natriuretic peptide, uric acid, soluble interleukin-2 receptor, and beta-2 microglobulin, also play a role in the diagnosing cardiac involvement.^[105,107,111]^ Accurate pathological diagnosis remains the gold standard for confirming cardiac involvement. Historically, this required a thoracotomy; however, less invasive procedures are now available, including TEE-guided biopsy, percutaneous endomyocardial biopsy, and pericardial fluid sampling.

Patients with cardiac involvement generally experience rapid disease progression, a poor prognosis, and high mortality, particularly those who present with ventricular tachycardia and heart failure as primary manifestations.^[107,112,113]^ Cytoreductive surgery, combined with adjuvant radiotherapy, has been reported to alleviate heart failure symptoms and improve patient outcomes.^[105]^ It is advisable to use non-invasive imaging techniques for the regular heart evaluations to enable the early detection of myocardial infiltration, even in asymptomatic patients with MF.^[105,107]^

### 2.6. Kidney

Upon autopsy, renal dissemination is observed in 24 to 31% of patients with MF.^[14,16,73]^ In the autopsy study conducted by Rappaport et al,^[16]^ among the kidneys of 14 patients exhibiting microscopic renal involvement, 11 were found to contain circumscribed tumors. The gross appearances of these tumors demonstrated significant variability, ranging from a small, unilateral, solitary, intracortical, subcapsular nodules to extensive, irregular, bilateral masses that extended from the cortices through the medulla into the renal pelvis. The nodules were typically distinct, well-circumscribed, and had a white, homogeneous cut surface, although occasional hemorrhages were also noted.

Renal involvement in MF has been documented with manifestations such as proteinuria, oliguria, acute renal failure, hypotension, and azotemia. It is worth noting that, in cases of MF with renal infiltrates, the characteristics and potential mechanisms of acute renal failure may exhibit variability.^[114]^

Imaging modalities are essential for diagnosing renal MF, given the nonspecific nature of its clinical presentation. Ultrasonography and CT are particularly valuable for both diagnostic purposes and assessing obstruction. The typical sonographic appearance is characterized by hypoechoic masses.^[114]^ The definitive diagnosis of renal MF is established through histopathological examination and immunohistochemical analysis. Renal biopsy remains the gold standard for diagnostic confirmation, demonstrating high sensitivity and specificity. Histopathologically, renal involvement shows the characteristic pattern observed in other malignant lymphomas, notably interstitial infiltration with preservation of both tubules and glomeruli.^[16,115]^ In one case, the renal parenchyma was reported to be replaced by tumor cells, leading to the loss of its normal architecture.^[114]^

Furthermore, some studies have suggested an association between CTCL and glomerular disease, particularly IgA nephropathy, however, renal biopsies in these patients have not demonstrated infiltration by lymphoma cells, leaving the pathophysiological link uncertain.^[116–118]^

### 2.7. Bone marrow

Bone marrow (BM) involvement in MF is considered uncommon and is categorized as visceral involvement (M1).^[8,10]^ The incidence of BM involvement in MF patients remains a contentious issue. For example, a study by Salhany et al^[119]^ found that BM involvement in epidermotropic CTCL, including MF and Sézary syndrome (SS), was relatively common, occurring in 21% of cases even at the plaque stage. It was also associated with widespread disease dissemination and reduced survival time. Similarly, Tang and coworker observed that cases with BM metastasis exhibited marginally lower survival probabilities compared to those without BM involvement.^[23]^ Therefore, there is a perspective that BM involvement indicates a poor prognosis of MF.^[25]^

However, other studies have reported that BM involvement is rare, particularly during the early stages. Chen et al^[120]^ followed 53 MF patients and 7 SS patients over 6 years, with only one SS patient showing BM involvement which did not affect the treatment regimen. Further BM biopsies during follow-up showed no specific lymphoma infiltration, even in patients with advanced clinical stages. These findings suggest that BM biopsies may not be essential during the initial staging and subsequent follow-up of MF patients. Additionally, multivariate analysis has not established the independent prognostic significance of BM involvement.

The clinicopathological significance of BM involvement in patients with MF remains a topic of ongoing debate, particularly concerning its prognostic implications. Conducting a BM biopsy to confirm frank lymphoma in MF is generally a low-yield procedure unless there is concurrent evidence of blood or nodal disease.^[8]^ ISCL/EORTC recommends performing a BM biopsy in patients with MF and SS who exhibit B2 blood involvement or unexplained hematologic abnormalities.^[8]^ However, there is a lack of consensus within the medical community regarding the necessity of BM biopsies during follow-up.

### 2.8. Eye

The prevalence of ophthalmic metastasis in MF encompassing both periocular and intraocular manifestations, remains poorly defined. Reported frequencies vary from 2% to 37%, depending on the referral center or study design.^[121–124]^ Ocular involvement typically manifests in the advanced stages of the disease, but it can occasionally be the initial indication of MF, though this is rare.^[125]^

The eyelids are commonly identified as the most frequently affected site, exhibiting a variety of clinical manifestations. These include persistent erythematous scaly patches or plaques, tumors of varying sizes with or without ulcers, thickened eyelids, blepharitis, poikilodermatous changes, flaking, and madarosis.^[123,124,126]^ In most cases, eyelid involvement often occurs alongside other facial lesions of MF.^[124,127,128]^ Baykal and colleagues noted that erythematous scaly lesions were the most common presentation of eyelid involvement in MF, followed by tumors.^[124]^ Conversely, another study found that blepharitis (50%), thickened eyelids (37.5%), and flaking (25%) were the most prevalent clinical manifestations, in that order.^[129]^ Ectropion has also been described as a significant prevalent manifestation of ocular disease in these patient.^[121]^

Eyelid involvement typically occurs as a secondary manifestation of early-stage disease. Among patients with eyelid involvement, 75% were diagnosed with advanced-stage disease (stages IIB, IVA, and IVB).^[124]^ Nevertheless, there have been documented cases where isolated eyelid lesions have been documented either the initial presentation or a sign of recurrence of MF.^[130,131]^ Periocular involvement of MF is more commonly observed in older individuals and males.^[124]^ In a multicenter study conducted at eye cancer centers, 7 out of 8 patients with MF involving the eyelid were male, with a median age of 78 years.^[127]^

Although the majority of ophthalmic manifestations pertain to the eyelid, tumor cells can also directly infiltrate the ocular surfaces in severe cases. There are heterogeneous clinical manifestations of intraocular MF, including anterior chamber inflammation, serous retinal detachment, retinal infiltrates and hemorrhages, keratitis (both ulcerative and interstitial), optic nerve infiltrates, uveitis, and vitritis.^[41,132–136]^ The vitreous is most commonly affected.^[132,136,137]^ Patients may experience visual acuity changes and/or floaters. Busch et al^[138]^ present a distinctive case involving a patient with MF characterized by conjunctival involvement confirmed through biopsy and the formation of xanthoma on the cornea. Despite the absence of tumor cell infiltration in the corneal biopsy, the authors suggest that the keratitis-associated epithelial damage facilitated the migration of conjunctival vessels carrying malignant cells across the limbus, thereby seeding the exposed corneal stroma. Subsequently, the infiltration of malignant T-cells led to lipoprotein leakage, which, following tumor regression, was phagocytosed by macrophages, resulting in the development of xanthoma surrounding the tumor plaque.

The therapeutic approaches and response to ocular dissemination of MF are varied. Treatments such as intravitreal and/or systemic MTX, local radiotherapy, systemic chemotherapy, PUVA, total skin electron beam therapy, and monoclonal antibody therapy have been reported to have been reported for patients with ocular involvement.^[41,55,131,139]^

Ocular involvement generally indicates a poor prognosis in patients, with the exception of one multicenter study which reported that MF patients with eyelid involvement had a relatively favorable prognosis, evidenced by a 5-year disease-specific survival rate of 86%.^[127]^ Notably, in this study, only one patient exhibited a tumoral lesion.^[127]^ Among the documented cases, excluding those lost to follow-up and those with unspecified prognoses, all remaining patients with ophthalmic involvement ultimately succumbed to systemic disease progression, despite some experiencing significant alleviation of ocular symptoms and improvement in vision.^[133]^ Four cases of MF with intraocular involvement and immunophenotype conversion from CD4 + to CD8 + have been reported.^[41,132,135]^ The CNS was affected in 3 of these cases.^[41,135]^ This condition appears to be associated with more aggressive clinical behavior and is considered a precursor to intraocular and CNS involvement. Consequently, some researchers suggest that if a shift from CD4 + to CD8 + predominance is observed in skin biopsies, clinicians should maintain a high index of suspicion for potential intraocular disease and seek ophthalmological consultation, in spite of the absence of known extracutaneous manifestations.^[41,135]^

### 2.9. Nails

Nail alterations in CTCL have been infrequently documented. Such changes are observed in MF, typically at an advanced-stage.^[140,141]^ There are only a limited number of reports confirming that nail changes are directly attributable to tumor-specific infiltration.^[142,143]^ In a retrospective analysis by Ehsani et al^[144]^ involving 60 patients diagnosed with MF, 18 patients (30%) exhibited nail changes. However, among the 10 patients who consented to periungual skin biopsies from scaly and erythematous areas, none showed histopathological findings consistent with MF. This negative result may be due to the lack of nail bed and matrix samples during the biopsy process. It is hypothesized that nail alterations in MF are associated with the aberrant production of T-cells in the skin, leading to prolonged growth arrest and general nail impairment. This occurs independently of other factors and diseases that may influence nail growth, morphology, and appearance.

Typically, nail involvement affects multiple digits, encompassing both fingers and toes, although cases involving a single digit have also been documented.^[142]^ Nail MF often presents as a complex and deceptive clinical picture, with symptoms ranging from yellowish discoloration and nail thickening to onycholysis, onychomadesis, subungual hyperkeratosis, pterygium formation, onychodystrophy, and thinning of the nail plates.^[142,143,145]^ Nail changes in MF may also be influenced by factors such as systemic drug use, necessitating diagnostic tests such as histopathology and/or TCR gene rearrangement to establish a definitive diagnosis. Biopsies should include both the matrix and the nail bed.

The treatment of nail involvement in MF remains inadequately standardized due to the limited number of reported cases. Reports suggest that patients who underwent systemic treatment exhibited partial or complete amelioration of nail lesions. However, it is important to note that the nail involvement alone in CTCL should not be considered an independent criterion for initiating systemic treatment unless otherwise indicated.^[141]^ Other treatments include PUVA, chlorambucil, and radiation therapy.^[142,143,145]^

### 2.10. Skeletal muscle

Epstein et al identified skeletal muscle involvement in 7% of MF patients postmortem^[14]^; however, antemortem muscle involvement has been documented in only 4 cases. In all documented cases, the gastrocnemius muscle was the most frequently affected site.^[146–148]^ Clinical manifestations include subcutaneous masses, muscle swelling, induration, and tenderness upon muscle palpation. In one instance, muscle symptoms appeared before cutaneous manifestations, leading to a delayed diagnosis.^[147]^ Among the 3 patients described in the literature, 2 succumbed to aggressive disease shortly after muscle involvement was diagnosed.^[146,148]^ Only one patient achieved disease stabilization through a treatment regimen that included PUVA, pegylated interferon alfa-2a, UVB311, and topical steroids.^[147]^

Histopathological examination revealed a continuous neoplastic T-cell infiltrate extending from the epidermis through the dermis and subcutis to the underlying muscle.^[146–148]^ It has been postulated that the malignant cells in MF exhibit a tropism towards the skin and musculature, akin to the lymphocytic behavior observed in dermatomyositis.^[147,149]^ The underlying mechanisms driving this musculotropism remain elusive, necessitating further investigation into the homing markers present in musculocutaneous lymphocytic infiltrates.

### 2.11. Penile

Male patients are also at risk of developing genital involvement by MF. Penile involvement of MF is relatively rarely seen, with only a few reports currently documented in the literature.^[150–153]^ Kodama et al^[152]^ proposed that penile MF may indicate hematogenous dissemination of the disease, similar to other metastatic malignancies, due to the rich vascular supply in the penile region.

The rash may can appear either independently or concurrently with rashes in other body areas. Affected regions of the penis reported include the penile shaft, corpora cavernosa, and glans. Clinical presentations can manifest as psoriasiform plaques, painful ulcers, or non-tender masses, potentially accompanied by decreased urine flow or painful erections.

The biopsied skin lesions on the penis were all conclusively identified as MF, with 2 cases exhibiting LCT. Four cases underwent various treatment modalities. Topical 5% imiquimod cream for localized penile plaques showed favorable outcomes, although caution should be exercised due to potential skin irritation side effects.^[151]^ Schaufler et al^[153]^ documented the initial instance of systemic brentuximab (anti-CD30) targeted therapy for penile MF, resulting in CR. Possible adverse effects include neuropathy, leukopenia, and fatigue.^[153]^ Local radiation therapy can yield a prompt improvement in skin lesions, thereby halting disease progression and altering clinical presentation with minimal adverse effects. Low-dose (12 Gy in 4 fractions) local radiotherapy effectively decreased the size of the cavernous tumor; however, the patient experienced adverse effects in the form of erectile dysfunction and penile sensory disturbance.^[152]^ Another patient underwent a combination of surgical excision, local radiotherapy, and systemic chemotherapy, yielding positive outcomes that underscore the significance of a multidisciplinary approach to treatment.^[150]^

### 2.12. Thyroid and parathyroid glands

The involvement of the thyroid and parathyroid glands by MF has been predominantly documented in autopsy reports,^[14–16]^ with only a limited number of case reports available in the literature.^[154]^ In the study conducted by Rappaport et al,^[16]^ the thyroid was identified as one of the most frequently affected extracutaneous organs, with an involvement rate of 75%, followed by the lungs (75%), spleen (60%), liver (53%), and kidneys (44%). Additionally, parathyroid involvement was observed in 25% of the cases. In a study by Epstein et al,^[14]^ among 86 postmortem patients, 12 (14%) exhibited thyroid involvement, and 4 (5%) exhibited parathyroid involvement. In a 1974 study, Long and Mihm reviewed 15 autopsy cases of MF with extracutaneous involvement and identified thyroid involvement in 2 cases.^[15]^

There are no specific laboratory findings associated with thyroid lymphomas.^[155]^ Despite the absence of specific laboratory markers, thyroid ultrasound is the recommended initial imaging modality.^[155,156]^ CT and MRI provide superior delineation of disease extent. Additionally, 18-fluorodeoxyglucose (FDG) PET demonstrates increased uptake throughout the affected thyroid gland. PET scans offer enhanced diagnostic accuracy for staging and evaluating treatment response.^[157]^

### 2.13. Others

Furthermore, involvement of other organs such as the adrenal glands, thymus, ovary, vagina, fallopian tubes, and breast has been documented; however, these occurrences are rare and predominantly observed in autopsy studies. There are no specific clinicopathological features available for a comprehensive summary and review.

## 3. Conclusion

While MF predominantly affects the skin, numerous studies have shown that it can metastasize, with a 10% in patients at the patch/plaque stage and 35–41% in those presenting with tumors or erythrodermic involvement.^[6]^ The incidence of clinical reports highlighting extracutaneous involvement is significantly lower than that revealed in autopsy series, suggesting an underdiagnosis of such involvement. This underdiagnosis can be attributed to several factors. Firstly, the clinical manifestations of extracutaneous involvement are often subtle and challenging to distinguish from other pathologies affecting these sites. Secondly, in cases with extracutaneous involvement, which typically present with extensive cutaneous rashes, the more accessible rash is often chosen as the biopsy site rather than the affected extracutaneous organ. Moreover, extracutaneous manifestations usually appear in the advanced stages of the disease progression; however, they can also present as the initial or sole manifestation of MF, frequently leading to misdiagnosis or oversight. Lastly, there is a general lack of awareness among physicians regarding the manifestations of MF outside the skin.

Patients with extracutaneous involvement typically show a poor response to treatment and have a short-term prognosis. Nonetheless, appropriate treatment can alleviate symptoms and reduce tumor burden. On the other hand, excessively aggressive treatment might compromise the patient’s immune system, potentially accelerating mortality.

In patients with a history of MF who exhibit extracutaneous symptoms, it is imperative to consider the possibility of metastatic spread involving visceral organs as a potential complication and to promptly exclude this diagnosis due to its potentially devastating consequences. Advanced cutaneous manifestations, including tumoral lesions or erythroderma, along with significant lymph node involvement, LCT, or a shift in immunophenotype from CD4 + to CD8+, are indicative of an increased risk of progression to extracutaneous involvement. These conditions require heightened vigilance and prompt intervention by clinicians.

## Author contributions

**Writing – original draft:** Lingxi Liu.

**Writing – review & editing:** Xiaoyan Zhang.
